# Validated Ossicular Measurements on High-Resolution Computed Tomography (CT) in Live and Cadaveric Temporal Bones

**DOI:** 10.7759/cureus.22410

**Published:** 2022-02-20

**Authors:** Shraddha Jain, Sana Parveen, Sunil Kumar, Dhruv Talwar

**Affiliations:** 1 Department of Otorhinolaryngology, Jawaharlal Nehru Medical College, Datta Meghe Institute of Medical Science (Deemed to be university), Wardha, IND; 2 Department of Otolaryngology, Jawaharlal Nehru Medical College, Datta Meghe Institute of Medical Science (Deemed to be university), Wardha, IND; 3 Department of Medicine, Jawaharlal Nehru Medical College, Datta Meghe Institute of Medical Science (Deemed to be university), Wardha, IND

**Keywords:** middle ear modeling, ear ossicle, temporal bone, high resolution computed tomography, middle ear

## Abstract

Objective

The present study was undertaken to study the utility of high-resolution computed tomography (HRCT) temporal bone in live patients for providing the parameters of ossicular dimensions (OD), using standard assessment protocols, devised by the authors, with regard to the plane of CT scans and axes of measurement of ossicles.

Methods

This observational study was conducted from January 2021 to February 2022 at Acharya Vinoba Bhave Rural Hospital, Sawanghi, Meghe, Maharashtra, India, in the Department of Otorhinolaryngology and Department of Radiodiagnosis. HRCT scan was performed on temporal bones, 10 in live subjects with no otological complaints and 10 in cadaveric temporal bones. HRCT was also performed for all three ossicles after dissecting them out from the respective cadaveric temporal bones. Measurements of OD were taken using Universalmednet Software and vernier calipers and compared with published anatomic data.

Results

The measurements taken using HRCT scans of the live subject and cadaveric bone as well as the ossicles taken using calipers fall within the range of published studies. Slight variation is seen in the measurements of stapes and incus, stapes total height is 3.17mm, less than 3.3. Conventional HRCT temporal bone in live patients when performed under set circumstances can be adopted as a standard method for measurement of ossicular sizes for the generation of normative data for different races, ethnicity and sex.

Conclusion

The results of our study can have implications in terms of translational research as the measurements obtained can help in better understanding of middle ear biomechanics, prosthesis designing, developing ideal tympanoplasty methods in terms of size, shape and placement of autograft.

## Introduction

The tympanic cavity comprises auditory ossicles, namely - the malleus, incus, and stapes. These ossicles form a chain across the tympanic cavity from the tympanic membrane to the vestibule [1]. There have been many studies for the measurement of ossicular dimensions and various methods described include reflection microscopes [2], temporal bone dissection followed by measurement using vernier calipers [3] and X-ray Micro computed tomography (CT) scans [4]. The importance of these measurements lies in the construction of middle ear models for understanding auditory physiology and for middle ear reconstructive surgeries. There is no single standardized method of assessment. The present study was undertaken to study the utility of high-resolution CT (HRCT) temporal bone in live patients for providing the parameters of ossicular dimensions, using standard assessment protocols, devised by the authors, with regard to the plane of CT scans and axes of measurement of ossicles. CT was preferred in this study as it enables the direct measurement of the malleus, incus, and stapes and avoids the cumbersome process of drilling mastoids and dissecting out ossicles. This study has implications for providing a standardized method for the generation of normative data for different races and ethnicity, availability of ossicular dimensions for the purpose of three-dimensional (3D) reconstruction of the middle ear, in order to study middle ear physiology, for making 3D printed models of the ear for training purposes, and for ideal measurements of prosthetic implants and refashioning of the autologous grafts.

## Materials and methods

**Figure 1:**
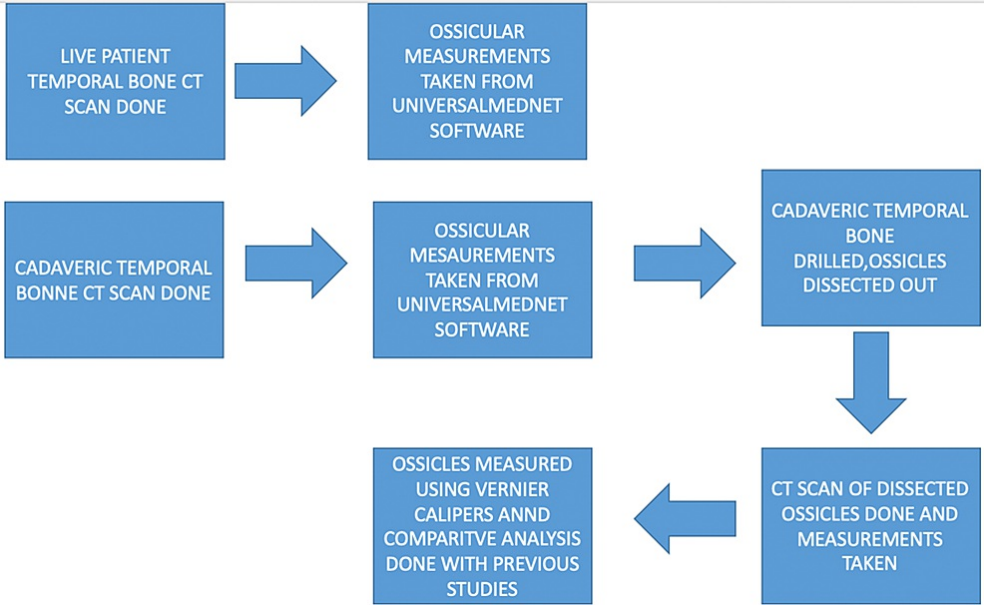
Flowchart depicting the methodology

The study has been conducted in the Department of Otorhinolaryngology and Department of Radiodiagnosis, from January 2021 to February 2022, at Acharya Vinoba Bhave Rural Hospital, Sawanghi, Meghe, Maharashtra, India, on middle ear ossicles, 10 from live adult males having no otological complaints and 10 from cadaveric temporal bones. The study was approved by the Institutional Ethics Committee, vide letter no. IEC/2020-21/9261 dated December 16, 2020. HRCT of the temporal bones was performed with 0.5mm cuts, having a specific emphasis on visualization of stapes, as it is the smallest bone in the body. Measurements were taken from the Universalmednet.com CT Software using the Measuring tool and simplified using diagrammatic representation for easier interpretation. Furthermore, the cadaveric temporal bone was drilled, the ossicles were dissected out and measured using HRCT as well as vernier calipers for validation. The CT scan machine used was - Seimens Somatom Scope Power - G-XL-81609. The cadaveric specimens used were formaldehyde preserved. A flowchart depicting the methodology of the study has been shown in Figure 1.

**Figure 2:**
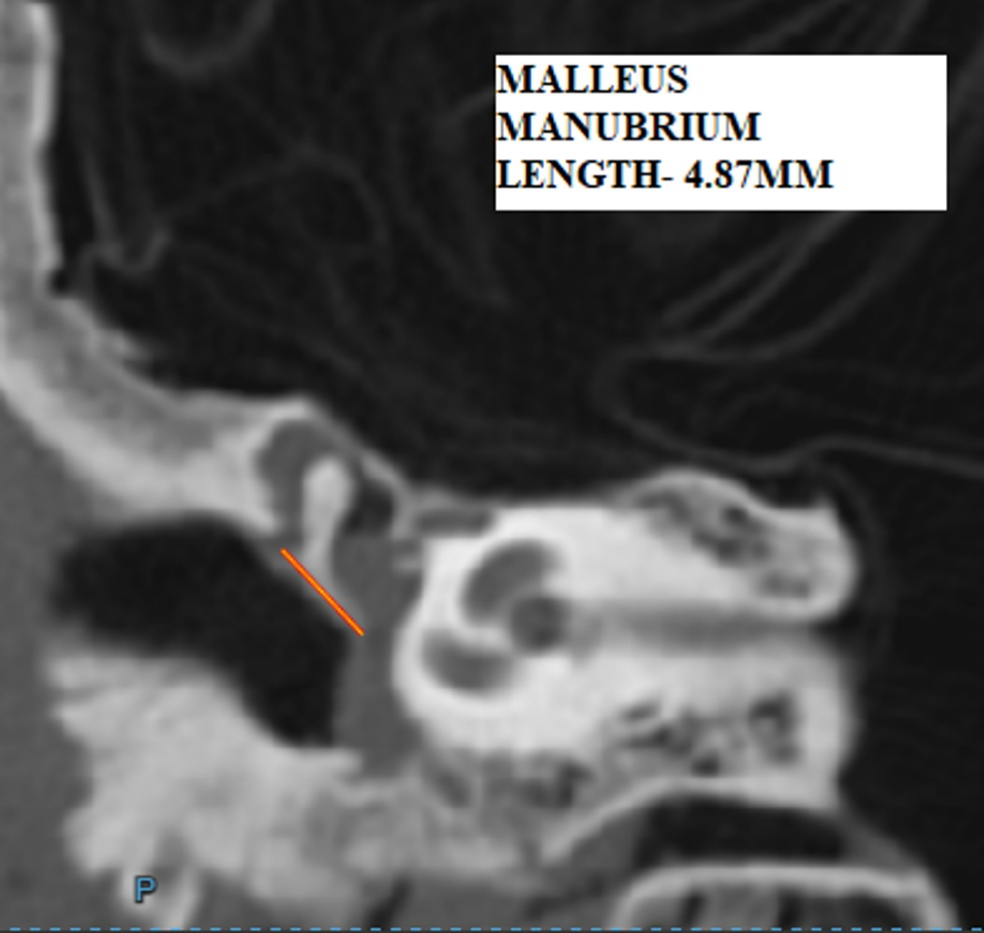
HRCT temporal bone - coronal section showing the length of manubrium – M1 - 4.89mm HRCT - high-resolution computed tomography

**Figure 3:**
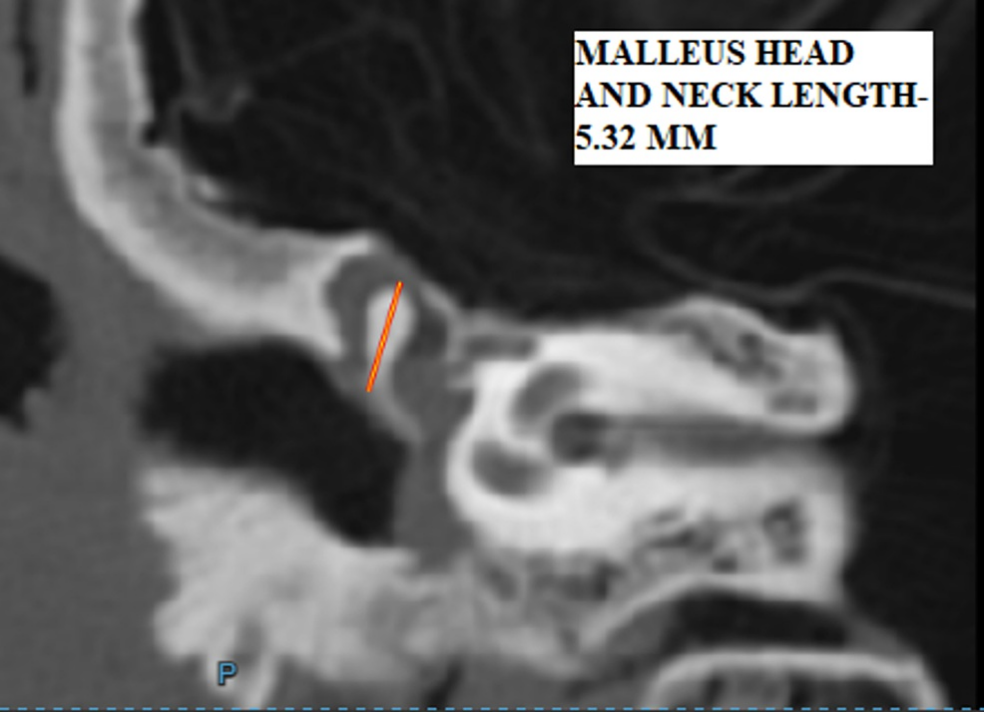
HRCT temporal bone - coronal section showing malleus - length of head and neck – M2 - 5.35mm

**Figure 4:**
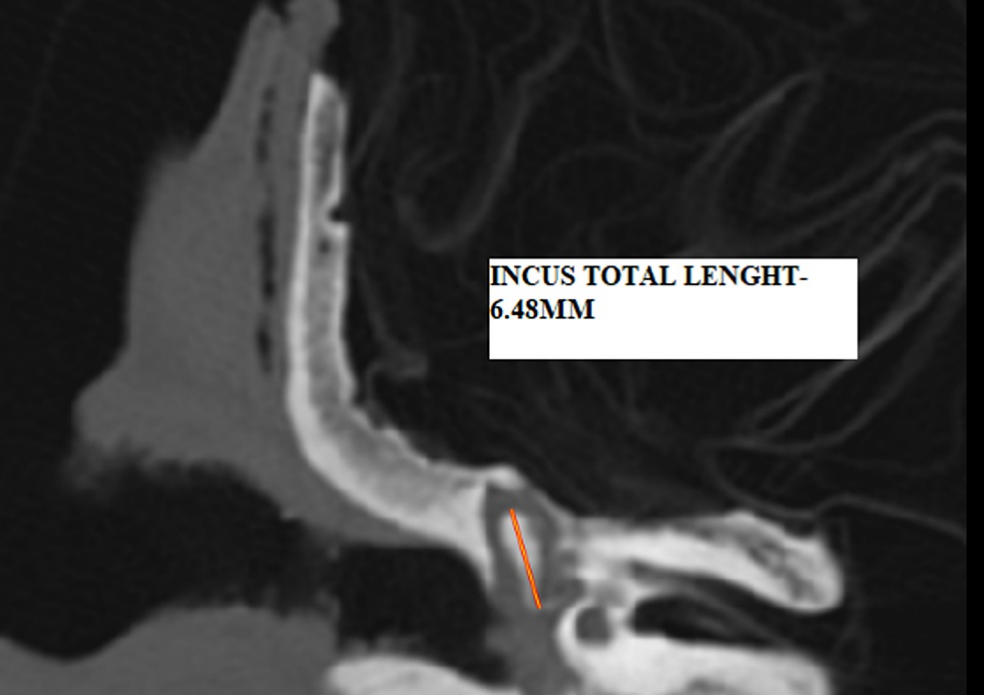
HRCT temporal bone - coronal section showing the total length of incus – I1 - 6.5mm HRCT - high-resolution computed tomography

**Figure 5:**
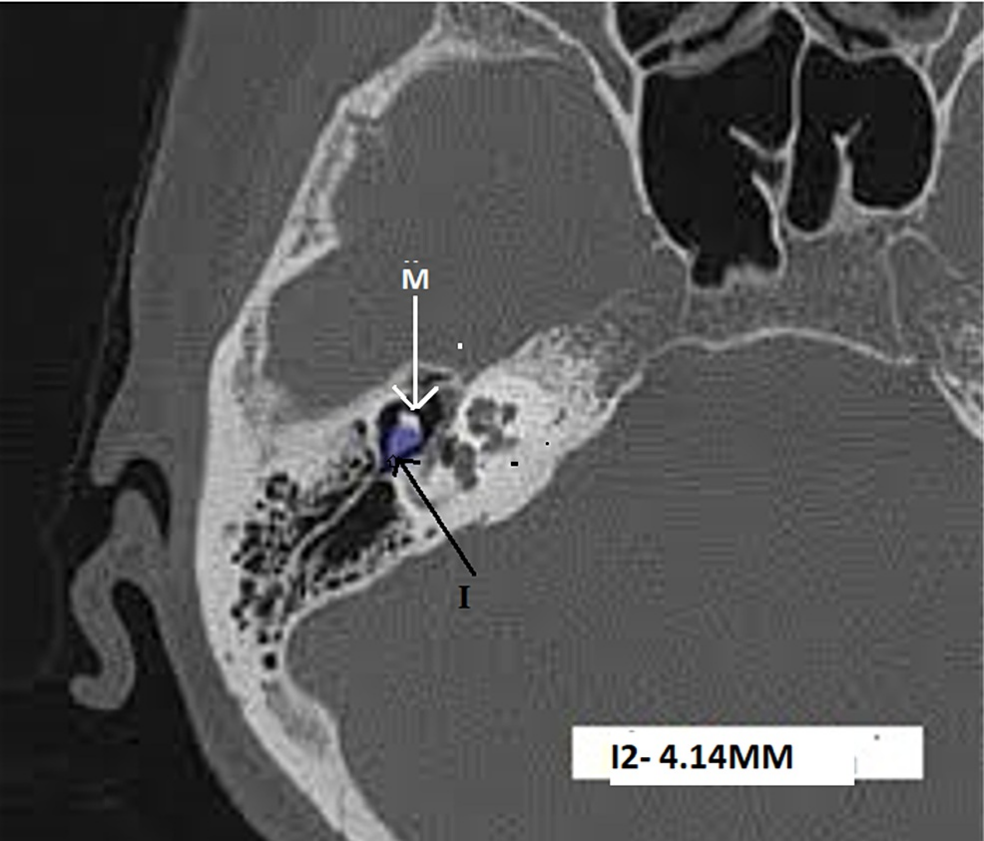
HRCT temporal bone - axial section at the level of ice-cream cone appearance of ossicles showing incus width – I2 - 4.15mm HRCT - high-resolution computed tomography, M - head of malleus, I - incus width

**Figure 6:**
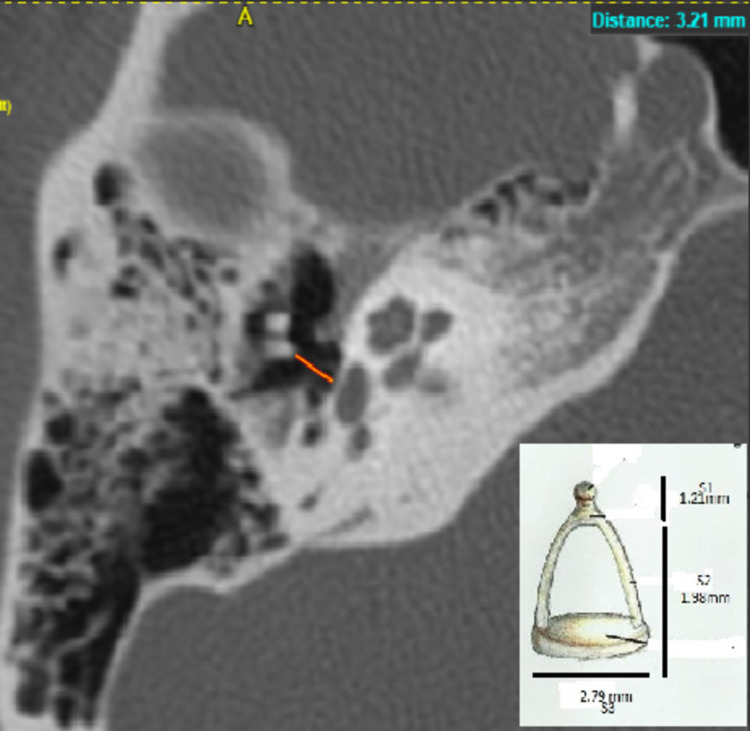
HRCT temporal bone - axial section showing stapes total height - 3.21mm HRCT - high-resolution computed tomography

The following measurements were taken:

1) Measurements of the malleus

a) Length of manubrium: distance from the end of the lateral process to the end of manubrium in the coronal section (Figure 2).

b) Length of head and neck: maximal distance between the top of the head and the end of the lateral process in the coronal section (Figure 3).

c) Width of the head of the malleus in the axial section.

2) Measurements of incus

a) Total length: maximal distance between the superior edge of the body and the end of the long process in the coronal section (Figure 4).

b) Total width: maximal distance between the superior edge of the body and the end of the short process in the axial section, at the level of “Ice Cream Appearance” (Figure 5).

3) Measurements of stapes

a) Head height: maximal distance between the top of the head and the neck in the coronal section (Figure 6).

b) Distance between neck and footplate in the coronal section.

c) Length of footplate: maximal length of the long axis of footplate in the axial section.

## Results

The HRCT temporal bone scans were done on 10 live subjects, 10 cadaveric temporal bones, as well as of the dissected ossicles from the cadavers. Our pre-defined ideal planes of measurement for different ossicular dimensions revealed accurate measurements of the malleus “manubrium” and “head and neck” in the coronal section, and malleus width in the axial section. The incus length is best computed in the coronal view, and width in the axial view at the level of “Ice Cream Cone Appearance,” as well as stapes “head height” and “distance between stapes neck and footplate” in the coronal view, along with the “length of stapes footplate” in the axial section. HRCT temporal bone by all three modalities for measurement of ossicular dimensions yielded results that were comparable with direct measurements made on dissected ossicles by vernier calipers.

All the mean parameters of the three ossicles were analyzed statistically by applying the Independent Sample T-test/Mann-Whitney U test. All the statistical calculations were performed using the software SPSS version 20 (IBM Corp., Armonk, NY, USA). The following parameters were recorded (Table 1). On comparison of the measurement of each parameter in a live subject with its left side of the respective bone in a cadaver, no statistically significant difference was observed.

**Table 1:** Ossicular measurements of live patient HRCT, cadaveric HRCT, and ossicular CT scan in the present study HRCT - High-resolution computed tomography

Structure	Present Study Live Patient CT Measurements, Mean ± SD (mm)	Present Study Cadaveric CT Measurements, Mean ± SD (mm)	Present Study Cadaveric Ossicular CT Measurements, Mean ± SD (mm)	Present Study Cadaveric Ossicle Vernier Caliper Measurements, Mean ± SD (mm)
Malleus - total Length (adding manubrium and head and neck length)	7.48 ± 0.045	7.60 ± 0.71	7.51 ± 0.02	7.52 ± 0.03
Length of manubrium	4.87 ± 0.07	4.64 ± 0.21	4.88 ± 0.09	4.91 ± 0.03
Length of malleus head and neck	5.32 ± 0.077	5.16 ± 0.07	5.28 ± 0.08	5.26 ± 0.06
Incus - total length	6.48 ± 0.041	6.57 ± 0.05	6.52 ± 0.06	6.52 ± 0.07
Incus - total width	4.11 ± 0.073	4.14 ± 0.05	4.14 ± 0.06	4.14 ± 0.02
Stapes - footplate length	2.74 ± 0.053	2.82 ± 0.08	2.80 ± 0.02	2.8 ± 0.02
Stapes - footplate width	-	-	1.51 ± 0.01	1.50 ± 0.02
Stapes total height	3.18 ± 0.030	3.17 ± 0.02	3.17 ± 0.05	3.15± 0.05

## Discussion

There are many clinical implications for measurements of ossicular dimensions. However, this has not received much attention. For ideal middle ear reconstructive surgery results, it is important to know the size of the grafts for ossiculoplasty, which could vary according to race and ethnicity, and hence customization is required. Also, for understanding middle ear biomechanics, time and again various models have been constructed [4] and still, there is a need for an ideal middle ear model. Most of the models have used published data for ossicular measurements [5,6]. There was a need to standardize the ossicular measurement process, so that accuracy would be achieved in understanding auditory physiology, and also for ideal middle ear reconstructions.

Numerous studies have used different ways of measurement of ear ossicles, middle ear, and tympanic membrane of cats, rodents [7] as well as human subjects. In the current study, various sections of CT scans were utilized in order to provide a standardized method of evaluation and measurement of the ear ossicles, focusing on the sections in which they are best visualized. Axial views have been used for incus width as well as for stapes footplate length. Coronal sections have proven beneficial for computing the length of malleus head, neck, and manubrium, incus length, the distance between stapes neck and footplate, and measuring the stapes head. This method has helped in forming a standardized way of measuring the ossicles that can prevent discrepancies and errors.

After an extensive literature search, we could identify a few studies which have measured middle ear ossicles by cadaveric dissection [8-11] and direct radiological measurement [9,4,12,13] and by analyzing morphometric variations among races [14], sexes [15], and ethnicities [16,17].

The advantage of the MicroCT is that a good 3D model of the ear can be constructed even with fewer CT sections and the ligaments can also be visualized [18]. These measurements can be applied in the designing of an ossicular prosthesis, graft measurements in tympanoplasty, and further development of models for analysis such as finite element modeling.

Our findings have been compared with published anatomic data (Table 2).

**Table 2:** Comparison of ossicular measurements by various studies using different methods of assessment

Parameter	Author, Year	Method of Assessment	Measurement (Mean +_ SD)
Malleus - Total Length	Sodhi et al., 2017 [10]	100 cadaveric temporal bones opened using chisel hammer, ossicles isolated and measured with vernier calipers	6.04-9.18 ± 0.54 mm
	Vinaychandra et al., 2014 [11]	25 cadaveric temporal bones opened using chisel hammer, ossicles isolated and measured with ECG recording paper	7.45 ± 0.39 mm
	Rathava et al., 2014 [19]	60 temporal bones opened using chisel and hammer, ossicles isolated and measured using Vernier Calipers	7.81 mm
	Jain et al., Current study 2021	Live subject HRCT Done, Cadaveric HRCT done, Cadaveric bone dissection and ossicular CT done followed by Measurement with Vernier Calipers	7.48 ± 0.045 mm
			7.60 ± 0.71 mm
			7.51 ± 0.02 mm
			7.52 ± 0.03 mm
Incus - Total Length	Sodhi et al., 2017 [10]	100 cadaveric temporal bones opened using chisel hammer, ossicles isolated and measured with vernier calipers	5.32-7.33 ± 0.42 mm
	Singh et al., 2015 [20]	120 incus isolated after manual dissection of cadaveric temporal bone, measured using digital vernier calipers	6.68 ± 0.49 mm
	Jain et al., Current study 2021	Live patient HRCT done, Cadaveric HRCT done, Cadaveric bone dissection done and ossicular CT done followed by Ossicluar measurement with vernier calipers	6.48 ± 0.041 mm
			6.57 ± 0.05 mm
			6.52 ± 0.06 mm
			6.52 ± 0.07 mm
Incus - Total Width	Sodhi et al., 2017 [10]	100 cadaveric temporal bones opened using chisel hammer, ossicles isolated and measured with vernier calipers	3.67-5.76 ± 0.40 mm
	Singh et al., 2015 [20]	120 incus isolated after manual dissection of cadaveric temporal bone, measured using digital vernier calipers	5.11 ± 0.39 mm
	Jain et al., current study 2021	Live patient HRCT done, Cadaveric HRCT done, Cadaveric bone dissection done and ossicluar CT done followed by Ossicluar measurement with vernier calipers	4.11 ± 0.073 mm
			4.14 ± 0.05 mm
			4.14 ± 0.06 mm
			4.14 ± 0.02 mm
Stapes - Footplate Length	Sodhi et al., 2017 [10]	100 cadaveric temporal bones opened using chisel hammer, ossicles isolated and measured with vernier calipers	2.17-3.13 ± 0.26 mm
	Rathava et al., 2014 [21]	60 temporal bones opened using chisel and hammer, ossicles isolated and measured using vernier calipers	2.78 mm
	Jain et al., Current study 2021	Live patient HRCT done, Cadaveric HRCT done, Cadaveric bone dissection done and ossicular CT done followed by Ossicular measurement with vernier calipers	2.74 ± 0.053 mm
			2.82 ± 0.08 mm
			2.80 ± 0.02 mm
			2.8 ± 0.02 mm
Stapes - Total Height	Sodhi et al., 2017 [10]	100 cadaveric temporal bones opened using chisel hammer, ossicles isolated and measured with vernier calipers	2.91-4.07 ± 0.26 mm
	Rathava et al., 2014 [21]	60 temporal bones opened using chisel and hammer, ossicles isolated and measured using vernier calipers	3.3 mm
	Jain et al., Current study 2021	Live patient HRCT done, Cadaveric HRCT done, Cadaveric bone dissection done and ossicular CT done followed by Ossicluar measurement with vernier calipers	3.18 ± 0.030 mm
			3.17 ± 0.02 mm
			3.17 ± 0.05 mm
			3.15 ± 0.05 mm

The MicroCT machine is much more expensive and much heavier, as compared to a conventional CT scan machine, not available at every center [22,23]. To the best of our knowledge, we could not identify any other study which has described measurements according to the different CT scan sections and compared with the corresponding measurements with vernier calipers as well as the existing findings.

Limitation

The primary limitation of the present study is the small sample size of the study. The study was conducted in a single-center thereby lacking representation from the rest of the country. Therefore, further studies are indeed required in order to validate and generalize the results of our study.

## Conclusions

HRCT temporal bone in live patients when performed under set circumstances can be adopted as a standard method for measurement of ossicular sizes for the generation of normative data, for different races, ethnicity, and sex. The malleus length after computing the manubrium, head, and neck were best visualized in the coronal section; the width is measured in the axial section. Coronal sections were useful in computing the length of the incus, stapes head height, and distance between neck and footplate. Adequate visualization of stapes footplate length and incus width was achieved by axial section. All the measurements were then compared with ossicular CT scan and vernier caliper measurements and were found to be close to those computations, except for slight variation in the stapes total height and incus width. The MicroCT scan, being a very expensive software is not available at all centers; hence, the authors propose an alternative method of constructing a middle ear model using validation from HRCT scans of live and cadaveric temporal bones as well as from ossicular dissection. This study's results have implications in terms of translational research as the measurements obtained can help in better understanding of middle ear biomechanics, prosthesis designing, developing ideal tympanoplasty methods in terms of size, shape, and placement of autograft.
